# The mediating role of schadenfreude between malicious envy and bullying in school

**DOI:** 10.1038/s41598-025-34408-2

**Published:** 2026-02-03

**Authors:** Maximilian Hofleitner, Joy Muth, Flora Fassl, Marko Lüftenegger

**Affiliations:** 1 Department for Teacher Education, Centre for Teacher Education, University of Vienna, Vienna, Austria; 2 Department of Developmental and Educational Psychology, Faculty of Psychology, University of Vienna, Vienna, Austria

**Keywords:** Schadenfreude, Envy, Bullying, Secondary school, Social emotions, Mathematics and computing, Psychology, Psychology

## Abstract

Bullying in school is a multidetermined phenomenon that becomes even more complex with the increasing relevance of social status at the onset of middle adolescence. This age - between 13 and 17 - is characterized by substantial social, emotional, and cognitive developments, implicating two negative social emotions in bullying behavior: envy and schadenfreude. Envy can be distinguished between benign envy, which involves improvement motivation, and malicious envy that leads to hostile feelings towards the envied person. Schadenfreude is defined as joy over the downfall of a rival. We propose that malicious envy is positively and benign envy negatively linked to bullying perpetration and that both effects are mediated by schadenfreude. In a preregistered study with a sample of *n* = 2,172 secondary school students, we collected data across two waves to test this proposed mediation model. Neither malicious nor benign envy is directly related to bullying; however, the effect of malicious envy on bullying perpetration is fully mediated by schadenfreude. Exploratory analyses suggest that these pathways differ by gender, indicating that in girls, malicious envy only affects bullying through schadenfreude, whereas in boys, bullying is likely influenced through other processes.

**Supplementary Information:**

The online version contains supplementary material available at 10.1038/s41598-025-34408-2.

## Introduction

 Bullying perpetration in school has been recognized as a serious threat to students’ well-being^1^. Whereas younger adolescents who engage in bullying perpetration are not fully aware of the impact of their actions^2^, starting with middle adolescence, bullying perpetration becomes a tool to intentionally defend or gain social status within peer groups^3,4^. As social status becomes more relevant for adolescents, social comparisons with peers consequently increase as well^5^, a crucial prerequisite for social emotions. Importantly, upward social comparisons involve a feeling of relatively lower social status and fewer resources, making confrontation less feasible^6^. Social emotions might serve as a first indirect pathway for expressing status concerns, before direct aggression occurs. Especially envy and schadenfreude may represent crucial steps towards bullying perpetration.

Envy occurs as the consequence of upward social comparisons and, in its benign form, is related to improvement motivation and altruism^7,8^. Malicious envy involves hostile feelings towards an envied person and the wish to drag them down. Schadenfreude comprises the joy resulting from the reduced social status of an envied rival^9^. The hostile feelings that accompany malicious envy may serve to justify this joy about a rival’s loss in status^10^. Schadenfreude, in turn, could channel these feelings into aggressive action to further reduce the social status of an envied other^9,11^. Benign envy, however, may help to buffer against schadenfreude and, subsequently, aggressive behavior by shifting cognitive resources away from the envied person and towards self-improvement^8,12^.

While first evidence links malicious envy and schadenfreude to bullying perpetration^13,14^, our knowledge of the possible psychological mechanisms that underlie these findings remains limited. Furthermore, evidence from the school context is lacking, even though adolescents spend most of their time in this social setting. Based on social comparison theory^15^ and using a large sample of secondary school students, this study aims to close this gap and explores the role of both malicious and benign envy in the prediction of bullying perpetration, and whether these relationships are mediated through schadenfreude.

## Literature review

### Bullying perpetration in school

Bullying is commonly defined as an intended aggressive behavior towards another person that is characterized by repetition and a power imbalance – meaning that the same person is targeted repeatedly and they are unable to properly defend themselves against this aggression^16^. This behavior can manifest itself either in direct ways, such as physical violence or verbal abuse, or it is carried out indirectly through, for example, social exclusion or rumor-spreading via different channels, which aims to undermine the social standing of the victim^17,18^. Taking the form of cyberbullying, this aggressive behavior among peers can also extend to contexts outside of face-to-face interactions and beyond the oversight of, for example, school personnel, further contributing to the already high prevalence of adolescent peer victimization^19^. Although these prevalences vary greatly depending on the country^20^ and the study methods^21^, recent meta-analytic evidence suggests that, on average, 25% of adolescents are bullying victims, 16% engage in bullying themselves, and 16% are involved in both victimization and perpetration^22^. While involvement in bullying was found to have negative consequences for all these groups^22^, the consequences for bullying victims have been particularly well-documented and range from heightened school absenteeism^23,24^ and lower academic achievement^25,26^ to increased depression and anxiety^1^ as well as suicidal thoughts and behavior^27,28^. Longitudinal studies also suggest that many of these negative psychosocial and academic outcomes persist throughout the school career and well into adulthood^29,30^. There is also consistent evidence for gender differences in bullying perpetration that is stable across countries, suggesting that male adolescents more often act as perpetrators and are also, to varying degrees, more often victims of bullying than female adolescents^31^.

Prompted by these findings, numerous intervention programs were designed and conducted to help schools address bullying behavior and reduce its negative consequences for their students (for an overview, see^32^). Many of these interventions were generally successful at reducing the prevalence of bullying behavior^33^. However, studies found success to be strongly dependent on the age of the participants. While interventions for primary students show the best results, effectiveness drops sharply after 7th grade^3,16,34^. These findings may be linked to the substantial socio-emotional and cognitive developments that occur during middle adolescence, commonly defined as the age between 13 and 17 years^35^. In contrast to younger children, adolescents may have a higher awareness of the consequences of their actions. Indeed, studies show that deficits in social skills and cognition are noticeably weaker predictors for bullying behavior in adolescents than in children^2^. In turn, social status and social evaluations gain increasing relevance in middle adolescence^5,36^. This heightened sensitivity to social evaluation and a growing tendency for adolescents to engage in social comparison with other peers form the basis for competitive peer rivalries^5^.

A basic tenet of social comparison theory^15^ proposes that comparing oneself with others is necessary to gauge one’s own status. When we find ourselves lacking due to an upward social comparison, this may trigger corresponding emotional and cognitive responses, like the feeling of inadequacy and the motivation to improve oneself^37^. If, however, improvement is not within our control, this feeling of inadequacy might motivate hostile feelings or more direct compensatory behavior such as actions to reduce the status of others.

The social school environment, regularly imposing graded performance situations and fostering peer-based status competition, likely contributes to the development of competitive relationships among adolescents that result in frequent social comparisons. Whereas competitiveness that is focused on personal development is associated with prosocial behavior, competitiveness involving the pursuit of social goals (e.g., wanting to be better than others) was found to lead to more targeted aggression^38,39^. In line with this reasoning, studies found bullying perpetration to be intentionally utilized by adolescents for status gain within the social hierarchy of peer groups^3,4^. Indeed, classrooms with frequent social comparisons are linked to higher bullying perpetration^40^. Due to the unique vulnerability of adolescents to social evaluations^35^, it is likely that the factors that ultimately determine if a peer rivalry leads to self-improvement and altruistic actions or to bullying perpetration involve negative social emotions.

### Envy and bullying perpetration

In accordance with social comparison theory^15,37^ envy is defined as a negative affective state that results from an upward social comparison^41^. Envy is always related to an envy object; this may be a physical object that we desire, or it can be more abstract, such as a skill or a personality trait that we would like to possess. Although envy is a universal human experience, studies found that men and women are envious of different things^42^ and that envy is much more likely to be elicited by a person of the same gender^43^. Contemporary theories further distinguish between two forms of envy: malicious envy and benign envy^7,41^. Malicious envy is the result when we feel that the envied person is undeserving of the envy object and we are unable to attain it ourselves. The perceived undeservingness then provides a permission structure for hostility and aversion towards the envied person that may mark them as a target for future hostile actions^8^. Malicious envy is also characterised by the wish to drag the other person down and reduce their status^44^; a wish that may be realized through bullying actions. Benign envy, instead, occurs when we believe the envied person deserves the envy object and that we are able to obtain it as well. Benign envy consequently also involves improvement motivation to acquire the envy object and its focus lies not on the envied person, but rather on the self. As it is not accompanied by hostility and shifts attention away from the envied person, benign envy may, therefore, even help to refrain from hostile actions^44^. Research on the relationship between envy and bullying is rare, but one study with Japanese students found that malicious envy was indeed positively linked to bullying perpetration^14^. Although benign envy previously was found to increase altruistic behavior in adolescents^8^, it did not emerge as a protective factor against bullying. Instead, benign envy was found to have a weak and positive correlation to bullying perpetration^14^. As hostile feelings are more immediate affective reactions, and improvement motivation and altruistic attitude may take longer to manifest, these results could be explained by the cross-sectional design that this study employed. Over a longer time, benign envy may still function as a protective factor against bullying. Following social comparison theory, we would also expect that both envy forms - as different reactions to an upward social comparison - entail different behavioral tendencies. Schadenfreude, another social emotion that is related to envy, may play a crucial role in this process^41^.

### Schadenfreude as a possible gateway to bullying

Recent theoretical arguments propose that schadenfreude, traditionally described as one general emotion, may be distinguished into different forms^9^. One of these forms, rivalry schadenfreude, may be closely connected to the feeling of envy and can be defined as the joy that results from the reduction of the social status of a rival and the relative improvement of one’s own status^9^. While rivalry schadenfreude is defined as the *passive* enjoyment of another’s downfall, if expressed publicly, it may manifest as relational aggression; a form of bullying. When directed at dominant others - e.g., an envied person - schadenfreude was found to play the role of a social-functional dominance regulator, reducing their social status, while increasing one’s own^11^.

When misfortune reduces the social status of a rival, whom we maliciously envy, schadenfreude is likely to be particularly strong^9,41^. Malicious envy can amplify this joy through its accompanying hostility that allows for positive feelings at events that would otherwise elicit sympathy or pity. Indeed, the stronger the aversion we feel towards an envied person and the greater the wish to drag them down, the more satisfaction we derive from their loss of status^11,41^. However, if the status of the rival is not permanently reduced and the reason for feeling malicious envy persists despite intermittent schadenfreude - i.e., it remains an upward social comparison with the rival -, it is unlikely that these hostile feelings will cease. Instead, social comparison theory suggests that these feelings may then also be expressed in actions to affect change^15,37^. If the desired outcome is to reduce the envied person’s status more permanently or to elevate one’s own then this could be accomplished by expressing schadenfreude publicly^11^. As bullies commonly utilize different forms of aggression for their goals, schadenfreude may therefore function as a possible gateway to other forms of bullying. While research about schadenfreude is rare, one study indeed found a positive link between schadenfreude and bullying perpetration in adolescents, supporting this assumption^13^.

Although both envy forms arise from an upward social comparison, benign envy differs in its relation to schadenfreude as it involves a focus on self-improvement rather than hostile feelings. Motivated by the wish for improvement, benign envy should therefore lead to behavioral tendencies that are distinct from malicious envy and that may act as a buffer for schadenfreude. Benign envy is also associated with altruistic tendencies^7,8^, which may suppress schadenfreude and therefore reduce bullying perpetration. Meta-analytic findings with adults imply modest support for this hypothesis, showing either a slightly negative link between benign envy and schadenfreude or no link at all^41^. Although there is some empirical evidence for our assumptions, these studies also used non-experimental cross-sectional designs and investigated both emotions separately, which precluded any examination of possible mediation mechanisms between them. Individual studies about schadenfreude with adults that examined gender also found differences between male and female participants, which may influence these processes as well^45^.

### The present study

**Fig. 1:**
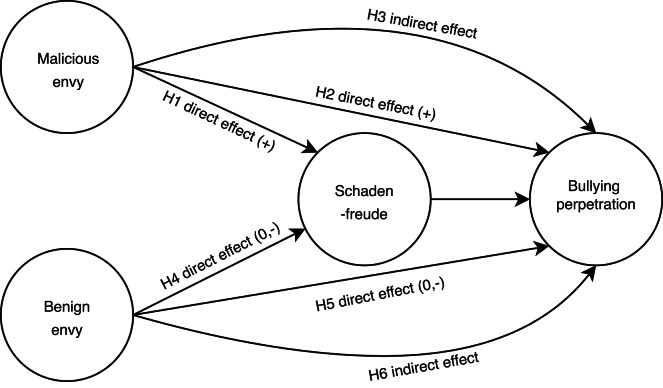
Our model proposes that the effect of malicious and benign envy on bullying perpetration is mediated by schadenfreude.

The aim of the present study is to further investigate possible antecedents of adolescent bullying perpetration by examining the role of envy and schadenfreude. Research on social emotions and their relation to bullying is scarce, and previous findings only examine both emotions separately, although there are strong theoretical reasons to believe that they affect bullying perpetration through different paths^15,37^. We propose a mediation process through which the effect of malicious and benign envy on bullying perpetration is mediated by schadenfreude (see Fig. 1). Our first research question is concerned with how malicious envy is related to schadenfreude and bullying. We suggest that malicious envy positively predicts schadenfreude (Hypothesis 1) and bullying perpetration (H2) and that the latter effect is mediated by schadenfreude (H3). Our second research question is focused on how benign envy is related to schadenfreude and bullying. Although we have strong theoretical assumptions, the empirical evidence about benign envy and its correlates and consequences remains limited and somewhat mixed. While most studies highlight its benevolent effects^7,46,47^, other findings suggest malevolent effects of benign envy^14,48^. Therefore, we expect that benign envy is negatively related to schadenfreude (H4) and bullying perpetration (H5). As the current empirical evidence is ambiguous - showing a weak negative correlation or no correlation to schadenfreude at all^41^ - we acknowledge that a negative correlation may be small or even become statistically non-significant. Similar to malicious envy, we also hypothesize that the effect of benign envy on bullying perpetration is mediated by schadenfreude (H6).

Our third exploratory research question is concerned with examining possible gender differences; as there are few previous findings about gender differences with respect to social emotions and bullying perpetration processes, we refrain from specifying any hypotheses. All research questions and hypotheses have been pre-registered at the Open Science Framework (OSF, https://osf.io/aemq2).

## Methods

### Sample and procedure

The data used in this longitudinal study was collected as part of a research project on social emotions in Austrian secondary schools. Secondary education in Austria starts after four years of primary school, and the academic track usually lasts eight years (four years at the lower level and four years at the upper level). Six schools participated in two waves, which were approximately six months apart (autumn 2023, spring 2024). A total of 1,554 students participated in the study at both waves, with additional 311 students participating only at wave 1 and additional 307 students participating only at wave 2. After data cleaning, we merged the data from both waves, leading to a final sample size of *N* = 2,172 participants who have participated in at least one wave. Participation was voluntary, and no compensation was given.

In both waves, students filled in an online survey during regular classroom lessons while being supervised by trained research assistants. As some items in the questionnaire refer to sensitive topics, during instructions, students were reminded of the confidentiality of their data and were encouraged to ask questions. At wave 1, students’ age ranged from 11 to 19 years with an average of 14 years (*SD* = 1.4). They attended grade seven (22.4%), eight (21.6%), nine (29.7%) and ten (26.3%), and female participants were slightly overrepresented (51.0%; male 46.9%; diverse 0.9%; did not want to disclose their gender 1.3%).

All students provided informed consent for participation and data processing. Students below the age of 14 were only allowed to participate with informed parental consent, which had to be provided beforehand. However, students aged 14 years or older did not require informed parental consent. In Austrian law, children between 14 and 18 are considered minors with limited legal capacity. As such, they are allowed to conduct legal transactions and consent to participation in (non-invasive) studies, for as long as they do not jeopardize the satisfaction of their basic needs. All parents and legal guardians of all students were therefore informed in detail about the study beforehand, and they were given sufficient time to provide informed parental consent or to object to the participation of their children. This study was conducted in accordance with the Declaration of Helsinki; all measurement instruments and the protocol were reviewed and approved by the Ethics Committee of the University of Vienna (reference number: 01014) and the regional Education Directorate. The study protocol was also approved by all participating schools.

## Measurement instruments

All items were answered with regard to general school experiences on a 5-point Likert-type scale, ranging from 1 (strongly disagree) to 5 (strongly agree) for benign envy, malicious envy, and schadenfreude, and from 1 (never) to 5 (almost every day) for bullying perpetration.

### Malicious and benign envy

Malicious and benign envy were measured at wave 1 using the Benign and Malicious Envy Scale (BeMaS^46^, which we adapted slightly to fit the school context. Both malicious envy (k = 5; sample item: “*Seeing the achievement of others makes me upset.*”, Ω = 0.83) and benign envy (k = 5; “*When I notice that my classmates are better at something than I am*,* I try to improve myself*”, Ω = 0.84) showed excellent composite reliability.

### Schadenfreude

Schadenfreude was measured at wave 1 using the subscale for rivalry schadenfreude from a newly developed measurement instrument tailored to the school context^47^. This scale (k = 5; “*My mood improves when the straight-A student gets a bad grade*”; Ω = 0.74) represents the schadenfreude resulting from upward social comparisons that are the necessary prerequisite for envy.

### Bullying perpetration

Bullying perpetration was measured at wave 2 using the short version from the PISA 2009 student questionnaire^48^ that was used in the *Social Motivation in the Classroom* study^38^. The scale consists of four items assessing different facets of bullying perpetration (i.e., physical attacks, verbal attacks, social exclusion, and cyberbullying) and showed satisfying composite reliability (Ω = 0.76). The item about cyberbullying was adapted by adding specific communication apps (e.g., WhatsApp) to facilitate understanding. All questions referred to the frequency of students engaging in the described behavior within the last two months to minimize recall bias and to avoid an overlap with the end of the first school semester and the school holidays that followed it.

### Statistical analyses

For all statistical analyses, we used R version 4.5.1. Using a tool for Monte Carlo power analysis for mediation models, we also conducted an a priori power analysis. After specifying correlations between all variables that are in line with prior literature, our results showed that *N* = 560 is required to find a full mediation at α = 0.05 with 80% power. All planned statistical analyses were pre-registered (https://osf.io/aemq2*).*

In preliminary analyses, we conducted confirmatory factor analyses and calculated composite reliabilities to examine the quality of all scales. Because we collected data within the school context, we inspected item-level intraclass correlations (ICCs) to assess the nested data structure (individuals nested in classes). For our exploratory research question about possible gender differences between boys and girls, we assessed configural, metric, and scalar measurement invariance across gender for all scales and the measurement model. Guidelines^49,50^ suggest that, for measurement invariance to be assumed, changes in model fit indices should not exceed the following limits: CFI/TLI = 0.01; RMSEA = 0.015; and SRMR = 0.03.

To answer our research questions, we specified a mediated structural equation model (SEM) with two predictors (benign and malicious envy), one mediator (schadenfreude), and one outcome (bullying perpetration). Because we only collected data in two waves, we used data from wave 1 for both predictors and the mediator as their relationship is theoretically well-grounded^51^. For our outcome we used data from wave 2. For our exploratory analysis, we used a multi-group SEM by specifying the same mediated structural equation model as in the main analysis with gender as a grouping variable. To examine whether regression slopes significantly differed between male and female students, we conducted Wald χ² tests of equality constraints within the multi-group SEM.

All analyses were conducted using the Robust Maximum Likelihood (MLR) estimator for robust standard errors. Missing values on the item level were low and ranged from 0.5% to 2% for all scales. To account for the missing data, we used full information maximum likelihood (FIML). Goodness-of-fit for all models was examined via CFI, TLI, RMSEA, and SRMR. We followed the guidelines^52^ regarding typical cutoff scores for excellent and adequate fit to the data, respectively: CFI and TLI > 0.95 and 0.90; RMSEA and SRMR < 0.06 and 0.08. Coefficients around 0.10, 0.20, and 0.30 can be interpreted as small, moderate, and large effects^53^.

## Results

### Descriptive and preparatory analysis

Descriptive statistics, bivariate latent correlations, reliabilities, ICCs, and model fit indices for all scales are presented in Table 1. The CFA models demonstrated excellent fit for all scales. Averaged ICCs were negligible across all scales (< 0.03).

**Table 1:** Descriptive statistics, latent bivariate correlations, intraclass correlations (ICC, average per scale), composite reliability (Ω), and model-fit indices for all used measurement instruments. Note. Boldface indicates *p* <.01. Benign and malicious envy share their model-fit indices as they were included in one CFA as two different factors of envy. Robust (scaled) fit indices are reported. Ω = McDonald’s Omega composite reliability, χ² = chi-square test statistic, df = degrees of freedom, CFI = Comparative fit Index, TLI = Tucker-Lewis Index, RMSEA = root mean square error of Approximation, SRMR = Standardized root mean square Residual.

	1.	2.	3.	4.
1. Benign envy	-			
2. Malicious envy	**0.384**	-		
3. Schadenfreude	**0.254**	**0.801**	-	
4. Bullying perpetration	0.032	**0.253**	**0.295**	-
*N*	1800	1802	1827	1814
*M*	3.03	1.97	2.23	1.75
*SD*	0.98	0.85	0.95	0.79
ICC	0.009	0.016	0.012	0.023
Ω	0.84	0.83	0.74	0.76
χ²(df)	225.926(34)		2.821(2)	4.013(2)
CFI/TLI	0.964/0.953		0.999/0.998	0.998/0.994
RMSEA	0.062		0.018	0.030
SRMR	0.052		0.007	0.009

Assessing the scales’ measurement invariance across gender (male, female), scalar invariance was supported for schadenfreude, benign envy, and bullying perpetration, although CFI and TLI were at the threshold of acceptable change for the latter. These results indicate that both groups did not significantly differ in their interpretation of these constructs. For malicious envy, only metric invariance could be established. However, the full measurement model, including all scales, reached scalar invariance (see Supplementary Table S1 for a summary of all measurement invariance testing results).

### Envy, schadenfreude, and bullying

Direct, indirect, and total effects of the mediation analysis are presented in Table 2. The results show a statistically significant and large positive direct effect (β = 0.83, *p* <.001) of malicious envy on schadenfreude, supporting H1. The negative direct effect of benign envy on schadenfreude, although very small, is also statistically significant (β = −0.06, *p* =.028), supporting H4. Schadenfreude also significantly and directly predicted bullying perpetration, with higher values in schadenfreude leading to more bullying perpetration (β = 0.25, *p* =.003). Although the direct effects on bullying perpetration were in line with our expectations, they were non-significant for both malicious and benign envy (*p* =.369 and *p* =.129), contradicting H2 and supporting H5, respectively.

**Table 2:** Results of the mediation analysis. Note. Benign and malicious envy were specified as predictors, whereas Schadenfreude was used as a mediator, and bullying perpetration was the outcome variable. For direct and total effects, Wald-type confidence intervals (CI) were calculated, and for indirect effects, the Delta-method was used. Two-tailed p-values were used with an α-level of 0.05. B = unstandardized coefficient, β = standardized coefficient, SE = standard error, ME = malicious envy, BE = benign envy, SF = Schadenfreude, BUL = bullying perpetration.

Path	β	B(SE)	CI	*p*
Direct effects				
ME → SF	0.825	0.604(0.035)	[0.536, 0.671]	< 0.001
BE → SF	− 0.063	− 0.042(0.019)	[−0.080, − 0.005]	0.028
SF → BUL	0.248	0.312(0.106)	[0.105, 0.520]	0.003
ME → BUL	0.078	0.072(0.080)	[−0.085, 0.230]	0.369
BE → BUL	− 0.061	− 0.052(0.035)	[−0.120, 0.015]	0.129
Indirect effects				
	β	B(SE)	CI	*p*
ME → SF → BUL	0.204	0.189(0.064)	[0.064, 0.314]	0.003
BE → SF → BUL	− 0.016	− 0.013(0.008)	[−0.028, 0.002]	0.081
Total effects				
	β	B(SE)	CI	*p*
ME → BUL	0.283	0.261(0.040)	[0.182, 0.339]	< 0.001
BE → BUL	− 0.077	− 0.066(0.034)	[−0.132, 0.001]	0.053

In accordance with H3, we found a statistically significant indirect effect of malicious envy on bullying perpetration, mediated via schadenfreude (β = 0.20, *p* =.003). Contrary to H6, no significant indirect effect of benign envy on bullying perpetration mediated via schadenfreude was found (*p* =.081).

### Exploratory analyses on gender differences

Table 3 presents the results from the multi-group mediated structural equation model, grouped by gender. Regarding direct effects, we found statistically significant and positive effects of malicious envy on Schadenfreude for both genders, with large effect sizes for males (ß = 0.81, *p* <.001) and females (ß = 0.84, *p* <.001). For males, all other direct paths were non-significant. However, for females, the model revealed a small but statistically significant and positive effect of Schadenfreude on bullying perpetration (ß = 0.25, *p* =.028), as well as a very small negative effect of benign envy on Schadenfreude (ß = − 0.12, *p* =.002). For females, we found a small statistically significant and positive indirect effect from malicious envy on bullying perpetration via Schadenfreude (ß = 0.21, *p* =.027). As the total effect of malicious envy on Schadenfreude for females was statistically significantly different from zero (*p* <.001), this indicates complete mediation. Interestingly, all indirect effects were non-significant for males, but the total effect of malicious envy on bullying perpetration showed a small positive and statistically significant effect (ß = 0.27, *p* <.001). We found the same results when calculating the mediation model solely for male participants and comparing the results to a males-only model with malicious envy as the sole predictor. This, in combination with the moderate correlation between malicious envy and benign envy (*r* =.384), rules out the possibility of suppressing or collinearity effects from benign envy. These results suggest that although malicious envy is associated with bullying perpetration among males (as suggested by the total effect), the specific mechanism is more ambiguous in this group. It may operate through other unmeasured pathways or reflect insufficient power to detect the indirect pathway.

**Table 3:** Results of the exploratory mediation analysis. Note. Testing the regression slopes for significant differences between genders revealed that only the path from benign envy to schadenfreude differed significantly between males and females (Wald χ²(1) = 5.92, *p* =.015). Together with the significant direct path found for females and the non-significant direct path found for males, this suggests that benign envy was more strongly related to schadenfreude for females than for males. All other paths did not differ significantly across gender. For direct and total effects, Wald-type confidence intervals (CI) were calculated, and for indirect effects, the Delta-method was used. Two-tailed p-values were used with an α-level of 0.05. β = standardized beta, SE = standard error, CI = confidence intervals, ME = malicious envy, BE = benign envy, SF = schadenfreude, BUL = bullying perpetration.

Path	Male				Female			
Direct effects								
	ß	b(SE)	CI	*p*	ß	b(SE)	CI	*p*
ME → SF	0.805	0.589 (0.055)	[0.481, 0.697]	< 0.001	0.838	0.607 (0.044)	[0.521, 0.693]	< 0.001
BE → SF	0.012	0.007 (0.027)	[−0.045, 0.059]	0.782	− 0.119	− 0.087 (0.028)	[−0.142, − 0.032]	0.002
SF → BUL	0.242	0.348 (0.191)	[−0.026, 0.722]	0.068	0.246	0.262 (0.119)	[0.029, 0.496]	0.028
ME → BUL	0.076	0.081 (0.137)	[−0.189, 0.350]	0.558	0.132	0.102 (0.091)	[−0.075, 0.28]	0.259
BE → BUL	− 0.093	− 0.085 (0.054)	[−0.191, 0.021]	0.116	− 0.022	− 0.017 (0.043)	[−0.101, 0.067]	0.692
Indirect effects								
	ß	b(SE)	CI	*p*	ß	b(SE)	CI	*p*
ME → SF → BUL	0.195	0.205 (0.112)	[−0.015, 0.425]	0.068	0.206	0.159 (0.072)	[0.018, 0.03]	0.027
BE → SF → BUL	0.003	0.003 (0.009)	[−0.016, 0.021]	0.785	− 0.029	− 0.023 (0.013)	[−0.049, 0.003]	0.084
Total effects								
	ß	b(SE)	CI	*p*	ß	b(SE)		*p*
ME → BUL	0.271	0.286 (0.065)	[0.158, 0.413]	< 0.001	0.338	0.261 (0.047)	[0.169, 0.354]	< 0.001
BE → BUL	− 0.091	− 0.083 (0.054)	[−0.189, 0.024]	0.127	− 0.051	− 0.04 (0.042)	[−0.122, 0.042]	0.342

## Discussion

This study investigated the complex role of two social emotions - envy and schadenfreude - in bullying behavior during middle adolescence. Using social comparison theory as a guiding framework^15,37^ and considering the conceptualization of schadenfreude as a social-functional dominance regulator^11^, we proposed that the effect of benign and malicious envy on bullying perpetration may be mediated by schadenfreude. We furthermore examined possible gender differences for these processes through more exploratory analysis.

In line with prior research and our hypotheses, we found a significant and positive relation between malicious envy and schadenfreude (H1); however, we found no direct link between malicious envy and bullying perpetration, contradicting our hypothesis (H2). Instead, the effect of malicious envy on bullying perpetration was fully mediated via schadenfreude, supporting H3. Also consistent with our hypothesis, we found a weak negative relation between benign envy and schadenfreude (H4), but no direct or indirect associations with bullying perpetration, supporting H5, but contradicting H6.

In conjunction, these findings suggest that malicious envy does not directly lead to bullying perpetration but rather influences it only through the emotional experience of schadenfreude. While the hostility inherent to malicious envy allows us to derive pleasure from other’s downfall that may temporarily mitigate the feeling of inadequacy^54^, the upward social comparison remains. Schadenfreude, when displayed publicly, however, can undermine the dominant status of others and qualify as relational aggression; a type of bullying^11^. As the fear of the rival other is then dispelled, hostile feelings arising from malicious envy may then be expressed in actions^37,55^, such as other forms of bullying. This aligns well with social comparison theory^37^ and the idea that moral emotions and their regulation are central to aggressive and antisocial behaviors in adolescents^56^. These findings extend previous research and further support the argument of schadenfreude as a social-functional dominance regulator^11^ by indicating its role in the emergence of bullying perpetration among adolescents.

The weak negative relationship between benign envy and schadenfreude is in line with previous meta-analytic evidence^41^ and suggests that focusing cognitive resources on self-improvement when experiencing envy mitigates schadenfreude to a small extent. Although benign envy was a significant negative predictor for schadenfreude, this did not translate into a significant indirect effect on bullying. Statistically, this pattern is plausible because the indirect effect depends on the product of two paths and the direct effect of benign envy on schadenfreude was very small. Contrary to our hypothesis but consistent with previous research^14^, benign envy does not appear to play a direct role in bullying perpetration. This may be explained by benign envy shifting cognitive resources away from others towards the self. Although both envy forms result from upward social comparisons, the hostile other-focused feelings that define malicious envy and appear to motivate bullying perpetration are absent in benign envy^7^. Instead, it appears that the emotional reactions and, consequently, behavioral tendencies that arise from benign envy are primarily self-focused. At first glance, this seems contradictory to other studies linking benign envy to prosocial tendencies^8,57^. However, prosocial behavior resulting from benign envy may ultimately serve the goal of self-improvement (e.g., I help someone learn something, so that I understand it better myself) and status-improvement (e.g., I help someone to be liked more) and can, thus, not be considered truly altruistic^7^. Behavior resulting from benign envy may only involve prosocial actions when they closely align with the personal goal of “reducing the difference between the envier and the envied person”^58^. As adolescents might use bullying perpetration strategically to gain social status^3,4^, refraining from targeted aggression may compromise this goal. This interpretation is further supported by studies suggesting that benign envy is positively linked with Machiavellian behavior, which is characterized by flexible ethics and the employment of manipulative tactics in pursuit of one’s own goals^12^. Taken together, these findings imply that benign envy may regulate emotional responses but not necessarily constrain bullying behavior.

Our exploratory analyses further suggest gendered pathways towards bullying perpetration. In line with our main results, we found that, for girls, malicious envy strongly predicted schadenfreude, which in turn modestly increased bullying perpetration. This is consistent with research showing that schadenfreude is directly linked with bullying perpetration through subtle forms of relational aggression and social exclusion among peers^13^. Our results suggest that, when girls have a heightened tendency to experience forms of envy associated with hostility, they may be more prone to feel schadenfreude, contributing to increased bullying behavior. Bullying performed by girls, therefore, may not be driven directly by envy itself but more by its emotional consequences, particularly schadenfreude, adding to the empirical evidence that moral emotions play important roles in peer aggression^56^. We further found that in girls, a heightened tendency for benign envy reduced schadenfreude, which supports the idea that constructive forms of envy are associated with less hostile affect towards the envied person^7^. However, our data showed that this did not translate to decreased bullying perpetration.

In boys, a heightened tendency for malicious envy likewise predicted schadenfreude but not bullying perpetration, neither directly nor indirectly. Interestingly, although the link from schadenfreude to bullying perpetration was non-significant, the total effect of malicious envy on bullying perpetration was significant. This suggests that malicious envy in boys also predicts increased bullying perpetration, but through different mechanisms than in girls. Considering gender differences for specific forms of aggressive behavior (i.e., more “indirect”, social aggression for girls and more “direct”, physical aggression for boys^31^), malicious envy in girls may be more closely linked to bullying perpetration through relational and socially aggressive emotions such as schadenfreude. For boys, in contrast, malicious envy might lead to bullying perpetration through anger, competitiveness, or striving for dominance. Since these emotions and motives were not assessed in our study, this interpretation remains speculative. Benign envy also does not have any effect on schadenfreude in boys, while it reduces schadenfreude in girls. This may be due to a stronger focus on self-improvement in girls than in boys, who might be inclined to pursue self-improvement through other means.

Collectively, our findings suggest that envy fuels bullying perpetration in both genders but through different pathways: for girls, primarily through schadenfreude, and for boys, through alternative processes, potentially comprising instrumental or dominance-based aggression due to different social norms or peer dynamics^31^. From a practical perspective, our results suggest that interventions may need to be gender-sensitive: for girls, targeting emotion regulation and reducing schadenfreude could be effective, whereas for boys, strategies that address competitive or dominance motives may be more relevant.

### Limitations and future directions

This study provided valuable observations about the complex role of emotional processes for adolescents’ bullying behavior, but these findings are constrained by some limitations. Although we conducted a longitudinal study, the data for our predictor (envy) and mediator (schadenfreude) were both collected at wave one. Therefore, the specific temporal order, and consequently, the causal ordering of envy and schadenfreude, is only an assumption. However, we believe that our assumption is well-grounded in theory^15,37,44^: As malicious envy is defined by hostile feelings and its wish to drag the envied person down, schadenfreude at their subsequent misfortune can be considered the joy at a wish fulfilled^44^. Empirical evidence supports this and points to malicious envy as an antecedent for schadenfreude^41^. Another limitation is the lack of data for our outcome variable at wave 1, which is needed to control for the baseline in our mediation analysis. Without baseline control, however, indirect effects may be overestimated due to the unmodeled stability of our outcome (bullying perpetration). We reasoned, however, that bullying dynamics will take some time to manifest at the start of the school year, when the first wave was conducted, and that measurement at wave 1 would also likely be biased due to false recalls (e.g., from bullying behavior from last school year). As bullying perpetration is unlikely to be stable over time - due to e.g., changes in class composition and teaching staff, continuous developmental changes in adolescence, and longer holiday breaks throughout the school year - we have reason to assume that this bias is minimal. Nevertheless, a baseline control is still strongly recommended for all variables, when conducting a mediation analysis using data from only two waves, as mediation paths will invariably be over- or underestimated to some extent^59^ and cautious interpretation of the results is advised^60^. Moreover, we measured envy and schadenfreude as trait emotions rather than state emotions to consider general tendencies over longer periods; dynamic, state-level associations between envy, schadenfreude, and bullying that may provide valuable information on how these relations unfold, therefore, could not be considered. Moreover, we measured envy and schadenfreude as trait emotions rather than state emotions to consider general tendencies over longer periods; dynamic, state-level associations between envy, schadenfreude, and bullying that may provide valuable information on how these relations unfold, therefore, could not be considered. There are also specific aspects of the Austrian education system that may have an impact on the generalizability of our findings. Although class compositions sometimes change depending on which specialization the students choose (e.g., technical or language classes), students generally attend most classes with the same peers throughout secondary school. This consistency can help foster positive class identity and social cohesion, which in turn motivates pro-social behavior^61^, possibly acting as protective factors for envy and schadenfreude. In Austria every class also has a homeroom teacher, who carries special responsibility for their students and acts as the first point of contact in case of conflicts, which may lower the threshold to address serious issues before they escalate further. When class cohesion is lower and teacher responsibility is more diffuse, this in turn may lead to more social comparisons, stronger negative social emotions and more bullying among students. Regarding our exploratory analyses, the interpretation of gender differences should remain cautious. In males, the non-significant indirect effect of malicious envy might result from limited statistical power, as the male subsample was smaller than the female, and indirect effects are harder to detect given their skewed sampling distribution. Differences might also stem from measurement issues, as for malicious envy, no full scalar invariance across gender could be established. This may have attenuated path estimates in one group.

Although our study highlights the relevance of social emotions in bullying perpetration, the full picture remains incomplete. Other findings, for example, also implicate moral disengagement - the suspension of ethical standards to allow oneself to engage in transgressive behavior^62^ - as a possible mediator between malicious envy and unethical decision-making^63^. Moral disengagement was also found to be positively linked with schadenfreude^64^. As adolescents become increasingly aware of the consequences of their actions, unethical decision-making, such as actively engaging in bullying perpetration, possibly necessitates a detachment from common moral standards to justify transgressive behavior^3,36^. Future research should therefore consider moral disengagement as another link through which malicious envy leads to schadenfreude and, ultimately, to bullying perpetration. This could be investigated using a multi-wave study design, which would also allow for the examination of more causal relationships.

This study is the first, to our knowledge, that examines the link between envy and schadenfreude and how they both relate to bullying perpetration. As demonstrated by the results of our exploratory analyses, the emotional processes leading to bullying behavior are nuanced and involve many factors. As the results of any one study need to be interpreted cautiously and evidence-based practice in education demands high standards^65^, we can, therefore, only offer some first thoughts on the implication of our findings. Generally, our results align well with prior theoretical assumptions^15,37^, implying that envy and schadenfreude are only intermediary steps leading to bullying perpetration. By focusing on the origin of these emotions (i.e., social comparisons), future research may gain a better understanding of bullying perpetration among adolescents. This could be investigated further by, for example, measuring the frequency of social comparisons among students as a possible moderator variable and controlling for how teacher behavior might provoke peer-to-peer comparisons. School-based interventions may likewise benefit from addressing social comparisons as the possible beginning of bullying behavior. Detecting social emotions in practice nevertheless might help practitioners to better understand the complex classroom dynamics. As both, envy and schadenfreude, however, are also considered socially undesirable emotions, observing them directly can be difficult, as they may be expressed more implicitly than other emotions such as test anxiety. Schadenfreude, although it can be recognized in malicious smiles and laughter, may also be deduced by a lack of empathy, while malicious envy may be revealed by the expressed feeling of unfairness for another one’s accomplishment or even the articulated wish or demonstrated action to take away the envy object from envied others. Future research could also benefit from testing plausible alternative models: Using a three-wave cross-lagged panel model the temporal order of predictor, mediator and outcome can be modelled directly. Alternatively, a reversed mediation model could also be used as a robustness check for this assumption. Furthermore, future research should consider investigating the relationship of envy, schadenfreude, and bullying perpetration using state measures, for example, employing experience sampling, to better understand the temporal unfolding of said relationship. Considering moral disengagement as a mediator between malicious envy and bullying perpetration may also prove fruitful for future studies. Lastly, investigating the effect of malicious envy on different emotional responses (e.g., anger, competitiveness) and motives (e.g., dominance strivings) may also help shine light on the gendered pathways leading to bullying perpetration.

## Supplementary Information

Below is the link to the electronic supplementary material.

Supplementary Material 1

## Data Availability

This study is part of the larger project “*Envy and Schadenfreude in School*”. All measurement instruments and anonymized data will be made available under the project name through the Austrian Social Science Data Archive (AUSSDA; https:/aussda.at). The syntax that we used to analyze our data will be published on the Open Science Framework (OSF). Further information about the project can be found in its respective preregistration [https://osf.io/6xrdh]. Information about the data collection and analysis is made available in our preregistration [https://osf.io/aemq2]. For further information or data requests, please contact the corresponding author, Maximilian Hofleitner (maximilian.hofleitner@univie.ac.at).
