# Supplementary material for: The mediating role of schadenfreude between malicious envy and bullying in school
Source: Sci Rep. 2026 Feb 3;16:5410. doi: 10.1038/s41598-025-34408-2 (PMC12886858; doi:10.1038/s41598-025-34408-2)
Supplement: Supplementary file 1 — Supplementary Material 1 [file 41598_2025_34408_MOESM1_ESM.docx]

|  | **χ²** | **df** | **CFI** | **TLI** | **RMSEA** | **SRMR** | **AIC** | **BIC** |
| --- | --- | --- | --- | --- | --- | --- | --- | --- |
| BE (Configural) | 25.490 | 10 | 0.995 | 0.990 | 0.042 | 0.013 | 26,194.498 | 26,359.029 |
| BE (Metric) | 26.777 | 14 | 0.996 | 0.994 | 0.032 | 0.016 | 26,187.785 | 26,330.379 |
| BE (Scalar) | 106.702 | 18 | 0.993 | 0.992 | 0.037 | 0.021 | 24,099.791 | 24,220.484 |
|  | **χ²** | **df** | **CFI** | **TLI** | **RMSEA** | **SRMR** | **AIC** | **BIC** |
| ME (Configural) | 22.427 | 10 | 0.996 | 0.991 | 0.037 | 0.013 | 24,031.517 | 24,196.099 |
| ME (Metric) | 31.822 | 14 | 0.994 | 0.991 | 0.038 | 0.025 | 24,032.911 | 24,175.549 |
| ME (Scalar) | 106.702 | 18 | 0.969 | 0.966 | 0.074 | 0.043 | 24,099.791 | 24,220.484 |
|  | **χ²** | **df** | **CFI** | **TLI** | **RMSEA** | **SRMR** | **AIC** | **BIC** |
| BUL (Configural) | 13.510 | 4 | 0.988 | 0.965 | 0.068 | 0.017 | 18,912.255 | 19,043.947 |
| BUL (Metric) | 14.894 | 7 | 0.987 | 0.978 | 0.055 | 0.024 | 18,911.278 | 19,026.509 |
| BUL (Scalar) | 32,600 | 10 | 0.973 | 0.968 | 0.066 | 0.034 | 18,928.609 | 19,027.378 |
|  | **χ²** | **df** | **CFI** | **TLI** | **RMSEA** | **SRMR** | **AIC** | **BIC** |
| Model 1 (Configural) | 533.687 | 258 | 0.965 | 0.958 | 0.037 | 0.046 | 90,358.903 | 91,036.695 |
| Model 2 (Metric) | 554.299 | 272 | 0.963 | 0.959 | 0.036 | 0.048 | 90,357.232 | 90,955.948 |
| Model 3 (Scalar) | 664.867 | 286 | 0.953 | 0.949 | 0.040 | 0.051 | 90,453.352 | 90,972.992 |
|  |  |  |  |  |  |  |  |  |
| **Supplemental Table S1.** Model fit indices for the different levels of measurement invariance for all scales and the mediation model. Robust (scaled) fit indices are reported. BE = Benign envy scale, ME = Malicious envy scale, BUL = Bullying perpetration scale, χ² = chi-square test statistic, df = degrees of freedom, CFI = Comparative Fit Index, TLI = Tucker-Lewis Index, RMSEA = Root Mean Square Error of Approximation, SRMR = Standardized Root Mean Square Residual, AIC = Akaike Information Criterion, BIC = Bayesian Information Criterion. | | | | | | | | |

**Supplemental Material**
